# Transcriptomic Analysis of Self-Incompatibility in Alfalfa

**DOI:** 10.3390/plants13060875

**Published:** 2024-03-19

**Authors:** Lulu Li, Sinan Liu, Yulu Wang, Yangzhou Shang, Zhi Qi, Hao Lin, Lifang Niu

**Affiliations:** 1 Biotechnology Research Institute, Chinese Academy of Agricultural Sciences, Beijing 100081, China; 13121233299@163.com (L.L.);; 2 School of Life Sciences, Inner Mongolia University, Hohhot 010021, China; qizhi@imu.edu.cn; 3 College of Life Science, Shanxi University, Taiyuan 030006, China; w826713967@126.com

**Keywords:** alfalfa, pistils, self-incompatibility, transcriptomic analysis

## Abstract

Alfalfa (*Medicago sativa* L.) is an important forage crop worldwide, but molecular genetics and breeding research in this species are hindered by its self-incompatibility (SI). Although the mechanisms underlying SI have been extensively studied in other plant families, SI in legumes, including alfalfa, remains poorly understood. Here, we determined that self-pollinated pollen tubes could germinate on the stigma of alfalfa, grow through the style, and reach the ovarian cavity, but the ovules collapsed ~48 h after self-pollination. A transcriptomic analysis of dissected pistils 24 h after self-pollination identified 941 differently expressed genes (DEGs), including 784 upregulated and 157 downregulated genes. A gene ontology (GO) analysis showed that the DEGs were highly enriched in functions associated with the regulation of pollen tube growth and pollen germination. A Kyoto Encyclopedia of Genes and Genomes (KEGG) analysis indicated that pentose and glucuronate interconversion, plant hormone signal transduction, the spliceosome, and ribosomes might play important roles in SI. Our co-expression analysis showed that F-box proteins, serine/threonine protein kinases, calcium-dependent protein kinases (CDPKs), bHLHs, bZIPs, and MYB-related family proteins were likely involved in the SI response. Our study provides a catalog of candidate genes for further study to understand SI in alfalfa and related legumes.

## 1. Introduction

Alfalfa (*Medicago sativa* L.) is a nutrient-rich perennial herb containing various vitamins, minerals, crude proteins, and fiber. It is an important livestock feed crop around the world, with a reputation as the “Queen of forage” [1,2]. Alfalfa is vital to dairy farmers, who graze their cows on the plant and harvest it to make hay and silage feed [2,3]. In addition, alfalfa provides environmental services, preventing soil erosion by holding cultivated soil together with its extensive roots [2] and, like other legumes, converting nitrogen from the air into compounds usable for plant growth [2,3]. Three assembled alfalfa genomes have been published: “Zhongmu No.1”, “XinJiangDaYe”, and “Zhongmu-4” [4,5,6]. However, alfalfa is an allogamous autotetraploid (2*n* = 4*x* = 32) and is partially self-incompatible [7,8,9,10,11], which makes its chromosomes highly heterogeneous and hinders the assembly of a high-resolution genome. It is therefore still difficult to clone alfalfa genes or conduct molecular breeding.

Self-incompatibility (SI) is a widespread outcrossing system present in flowering plants, in which flowers reject pollen from the same individual [12,13]. According to the zone of rejection in the pistil, SI systems fall into three main categories: sporophytic self-incompatibility (SSI), gametophytic self-incompatibility (GSI), and late-acting SI systems (LSI). In SSI, the incompatible phenotype is dominated by the parental plant’s genome, and the SI process often occurs on the stigma surface, where pollen germination is generally disturbed. SSI is controlled by a single *S*-locus comprising several tightly linked genes that encode female and male determinants [14,15,16]. The female determinant is the S-receptor protein kinase (SRK), which is specifically produced in the mature stigma. The male determinant in the vicinity of SRK is S-locus protein 11 (SP11)/S-locus cysteine-rich protein (SCR), which show anther-specific expression. When self-pollination occurs, SRK interacts with SP11, triggering a phosphorylation cascade in the stigmatic papilla cells, which inhibits the germination of self-pollen [17,18,19,20].

In GSI, the incompatible phenotype is controlled by the pollen’s haploid genome, and the SI process occurs in the style. GSI is also manipulated by a single polymorphic locus (*S*-locus) that consists of stable gene pairs of female and male determinants. In GSI, the female determinant is S-locus ribonuclease (S-RNase), which is highly expressed in the pistil. The male determinants are S-locus F-box (SLF) and S-haplotype-specific F-box (SFB), which are specifically expressed in pollen. Past studies indicate that more than one gene encoding F-box proteins is linked to *S-RNase* [21,22,23,24,25]. After pollination, the pollen tubes germinate on the stigma and grow through the style. During this time, S-RNases enter the pollen tubes. In the case of compatible pollination, S-RNase is degraded by ubiquitination, and the pollen tubes extend normally through the style. By contrast, S-RNase that interacts with SLF is not ubiquitinated or degraded, and this results in the inhibition of pollen tube growth, in a process that is also called “S-RNase cytotoxicity” [12,26,27,28,29,30]. Another GSI mechanism has been identified in poppy (*Papaver rhoeas* L.), in which the female stigma-located determinant *Papaver rhoeas* stigma *S* (PrsS) interacts with the male pollen-localized determinant *Papaver rhoeas* pollen *S* (PrpS) in the case of SI. This triggers a rapid increase in calcium ions (Ca^2+^) and, within minutes, a phosphorylation cascade, resulting in programmed cell death (PCD) and the inhibition of pollen tube growth [31,32].

In LSI, which is often observed in woody perennial species, the pollen tubes usually reach the ovarian cavity but are unable to accomplish fertilization [12,13,33,34,35]. Compared with GSI and SSI, LSI is poorly understood. Transcriptomic analysis of pistils has revealed that LSI is closely associated with Ca^2+^ signaling, plant hormone signal transduction, ATP-binding cassette (ABC) transporters, the mitogen-activated protein kinase (MAPK) signaling pathway, reactive oxygen species (ROS) metabolism, PCD-related genes, ubiquitin-mediated proteolysis, and the phosphatidylinositol signaling system [36,37,38,39].

Fabaceae species are important for livestock nourishment, and some of them are self-incompatible. To date, two types of SI systems have been described in Fabaceae: the RNase-based GSI (rejection in the style) [40] and the LSI (rejection in the ovary prior to fertilization or in the first divisions of the zygote) [6,41,42]. Past studies have identified and characterized the S-lineage T2-RNase genes in the *Trifolium pratense*, *Medicago truncatula*, *Cicer arietinum*, *Glycine max,* and *Lupinus angustifolius* genomes, but there is no evidence that Fabaceae gametophytic self-incompatibility is determined by Rosaceae, Solanaceae, and Plantaginaceae *S*-RNase lineage genes [43].

As one of the most important perennial leguminous forage crops, alfalfa’s seed yield and molecular breeding are highly restricted by its SI characteristics. There is a marked reduction in seed setting following self-pollination as opposed to cross-fertility [44]. It has been observed that pollen tube growth is more rapid in cross-pollination than in self-pollination [6]. Cooper and Brink (1940) conclude that partial self-incompatibility is due only in part to restricted pollen tube growth [41]. However, SI in alfalfa is an elaborate process whose molecular mechanism is poorly understood [45]. In this study, we observed pollen tube growth before fertilization. The type of SI in alfalfa might be GSI combined with LSI. To gain a deeper understanding of the SI response, we conducted a transcriptomic analysis of self-pollinated pistils and provided a catalog of candidate genes to enhance our understanding of the molecular regulatory mechanisms of SI in alfalfa.

## 2. Results

### 2.1. Pollen Tubes Got to the Base of Ovary after Self-Pollination

**Figure 1:**
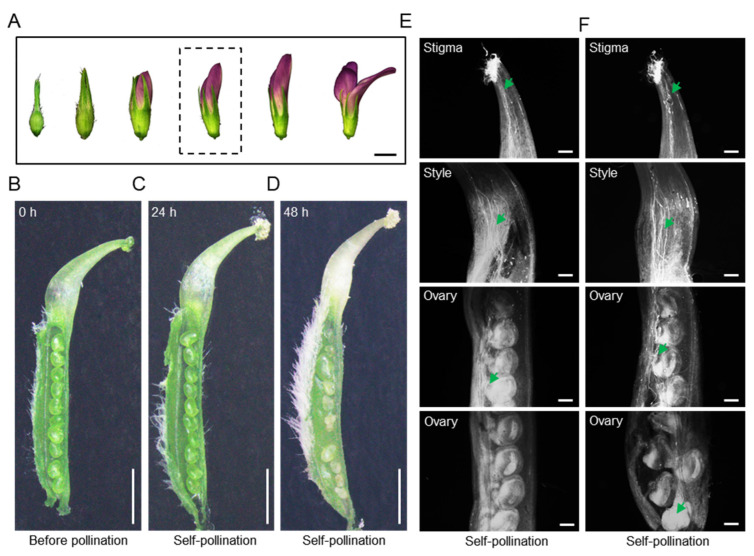
Pollen growth in pistils after self-pollination in alfalfa. (**A**) Different floral stages of alfalfa. Bar = 0.2 cm. The dashed frame indicates the stage used for the self-pollination experiment. (**B–D**) Dissected pistils at different stages: (**B**) before pollination, (**C**) 24 h after self-pollination, and (**D**) 48 h after self-pollination. Bars = 1 mm. (**E**,**F**) Aniline blue staining of pollen tubes (with stigma, style, and ovary shown) 8 and 24 h after self-pollination, respectively. Green arrowheads indicate pollen tubes. Bars = 100 μm.

To explore the molecular mechanism of SI in alfalfa, we focused on pollen tube growth after self-pollination. We used unopened but expanding flowers (fourth from the left in Figure 1A) for our study. Ovules of emasculated flowers with self-pollination, where pollen was collected from different flowers of the same plant, were investigated. We did not observe any obvious differences between the before-pollinated and self-pollinated ovules 24 h after pollination, but 48 h after pollination, many self-pollinated ovules had collapsed (Figure 1B–D). Specifically, 48 h after self-pollination, the mean number of developing ovules per flower was 1.6 ± 1.08, which was significantly less than that in cross-pollinated flowers (3.9 ± 1.73) (Figure S1, Table S1). The results indicated that partial SI was taking place in alfalfa. Aniline blue staining of the pollinated pistils showed that the pollen tubes had already entered the ovarian cavity at 8 h and had just reached the base of the ovarian cavity 24 h after self-pollination (Figure 1E,F). The number and fluorescence intensity of the pollen tubes in the pistils decreased gradually from the stigma to the ovary (Figure 1F).

### 2.2. Quality of the Alfalfa Pistil Transcriptome

The pistils were collected 24 h after either self-pollination (SP) or non-pollination (NP, emasculation without pollination) for transcriptomic analysis. High-quality reads from six RNA sequencing (RNA-seq) libraries were obtained after filtering out the low-quality reads. The Q30 quality score was more than 93.77% for each sample, and about 93.95% of the clean reads mapped to the reference genome “XinJiangDaYe” (Table 1), among which 164,632 protein-coding genes were predicted in the four sets of genomes [9]. To validate the accuracy of RNA-seq data, 15 DEGs were randomly selected and verified using quantitative real-time PCR (qRT-PCR; Table S2). The expression patterns of these genes were consistent with those determined using the RNA-seq data (Figure S2A–O).

### 2.3. Differential Expression Analysis

**Figure 2:**
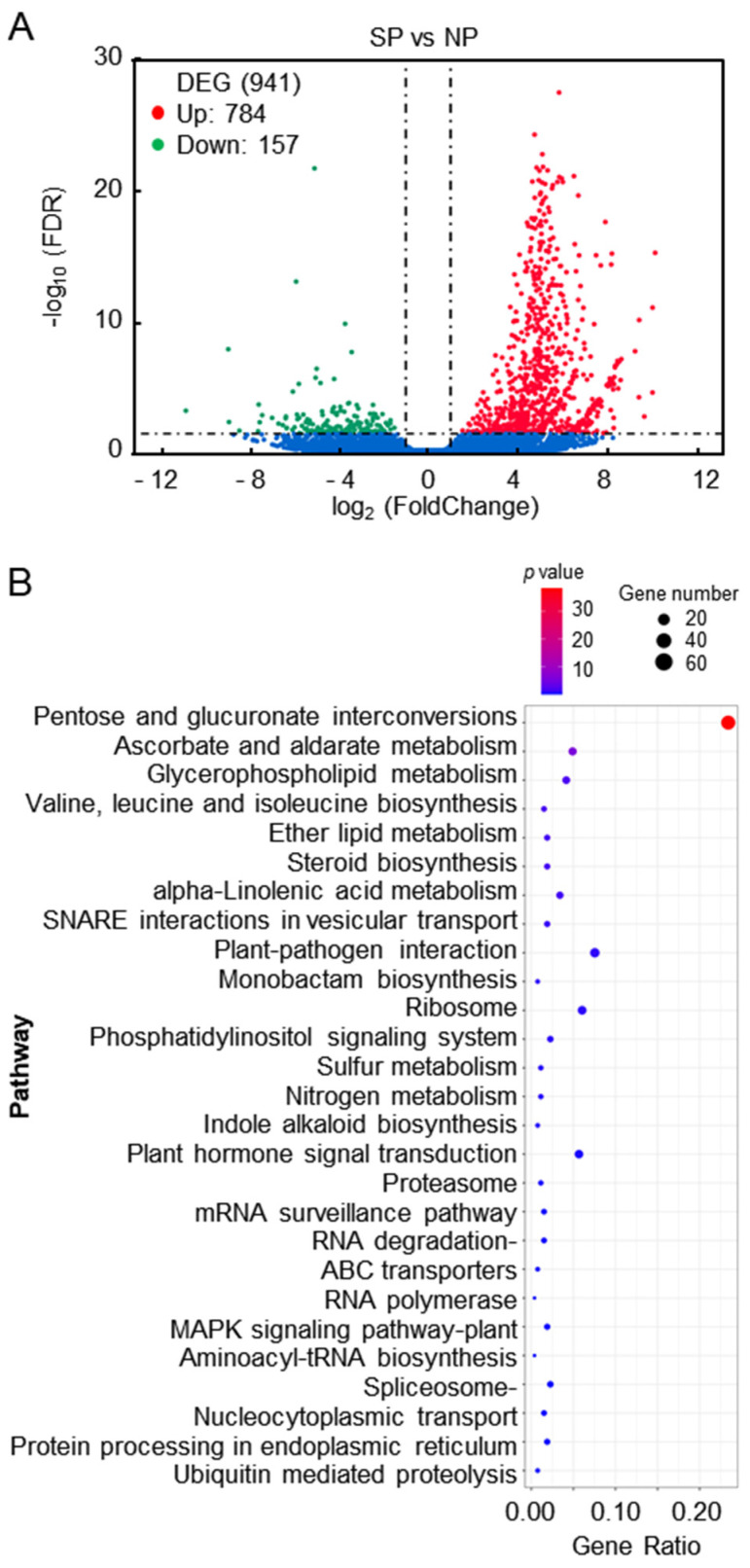
Analysis of DEGs in self-pollinated and non-pollinated pistils of alfalfa. (**A**) Volcano plot of all DEGs. (**B**) Significantly enriched KEGG pathways. The dot size represents the gene number, while the color represents the *p* value of the enrichment in each pathway. Gene ratio is the ratio of the number of DEGs annotated to the KEGG pathway to the total number of DEGs.

We next analyzed the differential expression of the alfalfa transcriptome during the SI process. We detected a total of 91,815 DEGs in the transcriptomes of the SP samples compared with the NP material. To ensure the accuracy of the DEGs, the screening condition was adjusted to incorporate both the false discovery rate (FDR) and the fold change (FDR < 0.05 and log_2_|fold change| > 1). Using this threshold, 941 DEGs were identified in the SP sample compared with the NP control, among which 784 were upregulated and 157 were downregulated (Figure 2A).

### 2.4. Functional Analysis of the DEGs

To further explore the possible association between DEGs and SI in alfalfa, we conducted a KEGG pathway analysis and a GO functional analysis. The DEGs were involved in 78 KEGG pathways, which were divided into five categories: metabolism, genetic information processing, environmental information processing, organismal systems, and cellular processes (Figure S3). The significantly enriched KEGG pathways included pentose and glucuronate interconversions (ko00040), the ribosome (ko03010), the spliceosome (ko03040), and plant hormone signal transduction (ko04075; Figure 2B). In addition, genes involved in the mitogen-activated protein kinase (MAPK) signaling pathway (ko04016), ATP-binding cassette (ABC) transporters (ko02010), plant–pathogen interactions (ko04626), and ubiquitin-mediated proteolysis (ko04120) were differentially expressed in the SP and NP samples (Table S3).

**Figure 3:**
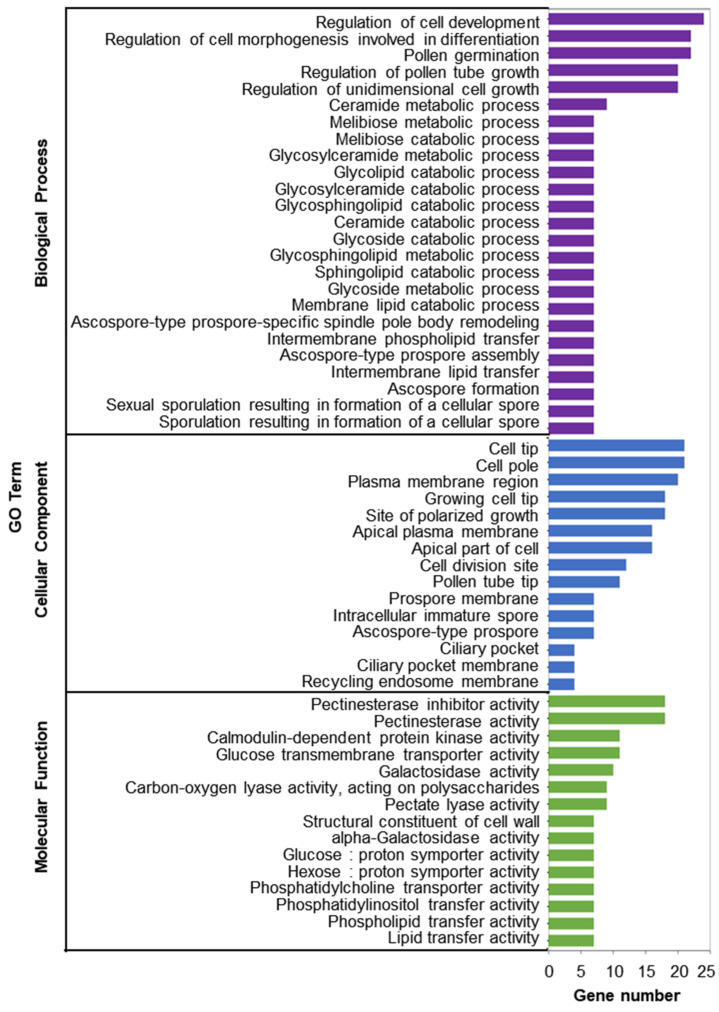
GO functional annotation of the pistil transcriptome during SI.

The DEGs were divided into 271 GO terms, including 187 biological processes (BPs), 28 cellular components (CCs), and 56 molecular function (MF) terms. In the BP category, the significantly enriched GO terms included the regulation of pollen tube growth and pollen germination, which would be logical for the SI response. The pollen tube tip term in the CC category and the calmodulin-dependent protein kinase activity in the MF category were also enriched in the SP DEGs and are probably associated with SI (Figure 3).

### 2.5. DEGs Involved in SI in Alfalfa

Based on the female and male determinants of SSI and GSI in other species, we searched for the homologous genes by annotation in alfalfa (https://modms.lzu.edu.cn/ (accessed on 11 March 2024)). We could not identify the homologs of SP11/SCR, S-RNase, PrsS, and PrpS in the DEGs; however, four F-box protein genes and 28 serine/threonine protein kinase genes were identified (Table S4). Ca^2+^ is a major secondary messenger that mediates signal transductions in various biological pathways, including pollination and fertilization [46,47]. In this work, nine genes related to Ca^2+^ signaling genes, specifically *CDPK* genes, were identified among the DEGs (Table S4).

**Figure 4:**
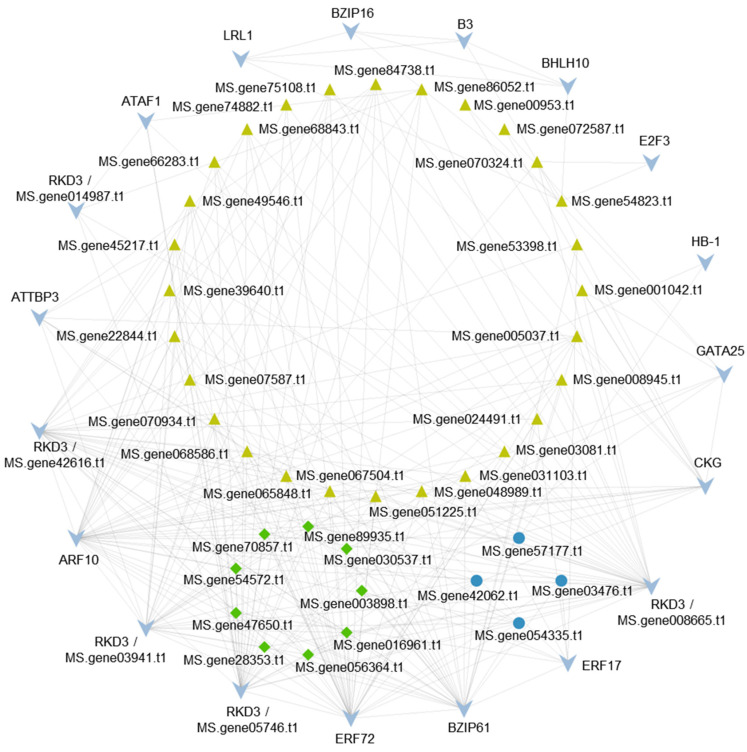
Gene co-expression network analysis.

In addition, transcription factors (TFs) are important regulators of multiple biological processes. We therefore searched for TF genes in the DEGs and identified bHLH, bZIP, and MYB family genes. The auxin and ethylene response factor genes were also present in the DEGs of the SP and NP comparisons (Table 2). Compared with NP, the expression levels of *BHLH10*, *ARF10*, and *ATTBP3* were significantly downregulated in the SP sample, while those of *BZIP61*, *RKD3*, *ERF72*, *ERF17*, *CKG*, *GATA25*, and *ATAF1* were highly increased (Figure S4, Table 2). By calculating the correlation coefficient between TFs and the genes mentioned above (|correlation coefficient| ≥ 0.8, *p* < 0.05), we conducted a gene co-expression network analysis, which showed that these TFs might regulate the expression of the DEGs mentioned above (Figure 4).

Light blue arrowheads represent TFs. Green rhombuses represent calcium-dependent protein kinases, blue circles represent F-box proteins, and yellow-green triangles represent serine/threonine protein kinases.

### 2.6. Analysis of Alternative Splicing in Alfalfa Pistils after Self-Pollination

**Figure 5:**
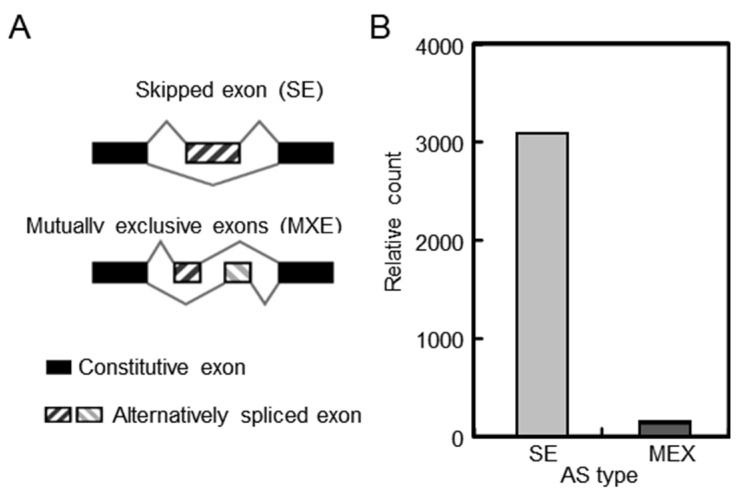
AS analysis of alfalfa pistils. (**A**) The AS types in alfalfa. (**B**) The relative count of different AS events detected in the transcriptome from SP compared with NP.

Alternative splicing (AS) is an important transcriptional regulation mechanism that could lead to the structural and functional polymorphism of transcripts and proteins. Based on different splicing modes, AS can be classified into five categories: skipping exon (SE), retained intron (RI), mutually exclusive exon (MXE), alternative 5′ splice site (A5SS), and alternative 3′ splice site (A3SS). In *Camellia oleifera*, the variable splicing events are involved in the LSI reaction [36]. Therefore, we conducted the AS analysis of SP compared with NP in alfalfa. Due to the limitation of alfalfa genome annotation, which lacked the 5’ UTR and 3’ UTR information of annotated genes, only MXE and SE were analyzed in the pistil of alfalfa during the SI reaction. As shown in Figure 5, 3226 AS events in SP were identified compared with NP, among which SE accounted for 95.6%. MXE only accounted for 4.4%. To further investigate the function of AS events in self-pollination, a KEGG pathway analysis was performed. The SE isoforms were enriched in ubiquitin-mediated proteolysis and RNA degradation, while the MAPK signaling pathway and plant hormone signal transduction were the pathways for the MXE-type transcripts. Among these differential AS events, approximately 35 serine/threonine protein kinase genes and 3 CDPK genes exhibited SE-type alternative splicing events, and F-box and *S*-RNase genes exhibited both SE-type and MXE-type alternative splicing events. The results suggested that variable splicing events were involved in the SI reaction.

## 3. Discussion

It has been reported that alfalfa shows partial self-incompatibility, and the elongation of pollen tubes in the style and ovary after self-pollination is slower than cross-pollination [6,41]. In this study, we observed that the pollen grains germinated on the stigma after self-pollination were less than those after cross-pollination, indicating that the pollen tube extension was partly hindered by the stigma, style, and ovary, respectively. Our GO function analysis showed that the SI response in alfalfa was related to pollen tube growth. However, the homologous genes of SP11/SCR or S-RNase that determine SI in other plant species were undetected in the DEGs, suggesting that the SI type in alfalfa might not be the reported SSI or GSI. Nevertheless, F-box protein genes and serine/threonine protein kinase genes were differentially expressed in SP and NP, while phylogenetic analysis indicated these F-box genes were not claded with previously reported *S*-pollen genes in the GSI of Prunus, Malus, Solanaceae, and Plantaginaceae [43]. Aniline blue fluorochrome staining revealed that most pollen tubes reached the ovary, but double fertilization remained unfinished, implying that SI in alfalfa also occurred in the ovary and might be LSI, which took place in the ovary prior to fertilization or in the first divisions of the zygote [42,47]. We observed that only a few pollen tubes reached the base of the ovary 24 h after self-pollination, which was less than cross-pollination (Figure S5), suggesting the fertilization process was hindered in the self-pollinated ovarian cavity.

The pollen tube grew slower after self-pollination than after cross-pollination. There might be some difference in the pollen tube tip. Small GTPases regulate diverse cellular processes, including signal transduction and cell polarity. Rho (Rac-Rop) GTPase accumulates at the apical plasma membrane of pollen tubes and regulates polar growth [48]. In the DEGs between the SP and NP samples, there were some GTPase genes, which meant that they might participate in SI response. In our study, we compared self- and non-pollinated pistils. It is thus possible that some of the DEGs are related to the plant’s response to the development of a normal pollen tube rather than to SI.

Ca^2+^ is a major secondary messenger and is crucial for pollen germination, pollen tube growth, and the fertilization process. In *Petunia inflate*, overexpression of Pi *CDPK1* caused a dramatic loss of pollen tube growth polarity, while increased expression of Pi *CDPK2* led to inhibition of pollen tube extension [46], suggesting CDPKs play an important role in pollen tube growth. In *Papaver rhoeas*, the incompatible stigmatic S-glycoproteins induced a transient increase in Ca^2+^ in pollen tubes, while no increase in Ca^2+^ was detected after the addition of compatible stigmatic S-glycoproteins, indicating that the SI response in *Papaver rhoeas* is mediated by Ca^2+^ [46]. Considering that CDPKs are important primary sensory receptors in Ca^2+^ signaling, further identification and characterization of CDPK genes within the DEGs between the SP and NP samples may help to elucidate the regulation of the SI response in alfalfa.

In *Brassica oleracea*, *SLG_2_* (*S* locus glycoprotein), related to the incompatibility phenotype, could produce the expected 1.6 kb transcript and an alternative 1.8 kb transcript. The two transcripts vary in different tissues, and the proteins they encode have potential implications for each other [49], indicating that AS is related to SI. In this study, 3226 SE-type and MXE-type AS events in SP were identified compared with NP, suggesting that AS might be involved in SI in alfalfa. Nevertheless, it is worth noting that *S*-RNases exhibited both SE-type and MXE-type alternative splicing events in our research, but their expression level showed no significant difference in SP and NP pistils.

TFs are important regulators of a huge variety of biological processes, including SI responses in plants [50]. In this work, 19 TFs that might be involved in the SI process were identified (Table 2). Among these TFs were RWP-RKs (possessing the RWPXRK motif), which can be classified into two subfamilies: NIN-like proteins (NLPs) and RWP-RK domain proteins (RKDs). NLPs function in nodule organogenesis and rhizobial infection, whereas RKDs participate in egg cell specification and differentiation or gametogenesis [51]. We also detected DEGs encoding GATA-type zinc finger proteins with TIFY domains, which control fiber development and participate in jasmonic acid signaling [52]. Other groups detected were the bHLH family and bZIP family genes, which play important roles in reproductive responses, stress responses, and signaling transduction [53]. The ARF, B3, and ERF gene families, associated with plant hormone signaling [54,55,56], were also observed in the DEGs; therefore, phytohormones might contribute to the SI response of alfalfa. Other DEGs, including the MYB-related family proteins, which regulate multiple developmental processes, such as pollen formation, seed germination, ovule fertilization, and seed formation [57,58,59], have been specifically shown to be involved in floral development and female reproduction [60,61]. In the self-pollinated alfalfa, the pollen tubes reached the ovarian cavity, but the ovules remained unfertilized, suggesting that SI might occur in the ovules where the *MYB* genes function. Taken together, our results showed that SI in alfalfa might be associated with F-box proteins, serine/threonine protein kinases, CDPKs, bHLHs, bZIPs, and MYB family proteins, providing a reference for future studies on SI in alfalfa and related legumes.

## 4. Materials and Methods

### 4.1. Plant Materials and Sample Collection

Alfalfa “Sanditi” (Barenbrug) was used in this work. After scarifying the seed coat with emery cloth, the seeds were germinated overnight in Petri dishes on moist filter paper at room temperature, then placed in the dark at 4 °C for 1 week. After this stratification, these seeds were transferred to a greenhouse at the Biotechnology Research Institute, Chinese Academy of Agricultural Sciences, Beijing, China, where they were grown under long-day (16 h light/8 h dark) conditions and 60% relative humidity at 25 °C. After two days, seedlings had germinated and were planted in mixed soil (soil/roseite = 1:2).

When one “Sanditi” plant was in full bloom, the unopened flowers, fourth from the left in Figure 1A, were emasculated and pollinated from 9:00 a.m. to 12:00 noon. The pistils were collected 24 h after self-pollination (with pollen from different flowers of the same plant) and non-pollination (emasculation without pollination) for the transcriptome. Three biological replicates were performed for each treatment, with 30 pistils in each replicate. All samples were immediately frozen in liquid nitrogen and stored at –80 °C until required for RNA extraction.

### 4.2. Aniline Blue Fluorochrome Staining of Pollen Tubes in Pistils

The pistils were collected 8 and 24 h after self- and cross-pollination and immediately placed in a fixing solution (95% ethanol/glacial acetic acid = 3:1) for 3 h or overnight if necessary. The fixed pistils were macerated in a 5 M NaOH softening solution for 24 h. After being rinsed with double-distilled water, the pistils were incubated in 0.1 M K_2_HPO_4_ (pH 10) buffer overnight. The next day, the pistils were transferred into a 0.1 M K_2_HPO_4_ (pH 10) solution containing 0.005 g mL^–1^ aniline blue (Solarbio, Beijing, China) for 3–4 h and then observed under a UV fluorescence microscope [62].

### 4.3. RNA Extraction and Transcriptome Sequencing

Total RNA was extracted from the SP and NP pistils using the TRIzol reagent (Thermo Fisher Scientific, Waltham, Massachusetts, USA) according to the manufacturer’s protocol. RNA concentration and purity were measured by NanoDrop 2000 (Thermo Fisher Scientific, Wilmington, DE, USA). The library construction was conducted using Hieff NGS^®^ Ultima Dual-mode RNA Library Prep Kit (Premixed version) (Yeasen, Shanghai, China). Firstly, mRNA was enriched from total RNA and fragmented. Secondly, the first-strand cDNA was synthesized using random hexamers, and then the second-strand cDNA libraries were created, end-repaired, and purified. Then, the libraries were amplified via PCR, and sequencing was performed using the Illumina HiSeq2500 platform in BerryGenomics (Beijing, China).

To obtain high-quality, clean reads, the raw reads were further filtered by fastp [63]. The filtering criteria were as follows: (1) reads with adaptor sequences were trimmed; (2) paired-end reads were removed when the number of N bases in a single-ended sequence read was greater than 5; and (3) low-quality reads were removed (the number of bases with quality value Q ≤ 15 accounted for more than 40% of the whole read). Relative expression levels of genes were estimated by the value of fragments per kilobase of transcript per million fragments mapped (FPKM). DEGs were designated using the DESeq2 method [64] with dual criteria of FDR < 0.05 and |log_2_foldchange| > 1.

### 4.4. GO Functional Enrichment Analysis and KEGG Pathway Enrichment Analysis

GO is a standard terminology system for describing gene function and is divided into three major categories: biological process (BP), molecular function (MF), and cellular component (CC). KEGG is a database resource for understanding high-level functions and utilities of the biological system, such as the cell, the organism, and the ecosystem, from molecular-level information, especially large-scale molecular datasets generated by genome sequencing and other high-throughput experimental technologies. We used clusterProfiler4.0 [65] to perform GO functional enrichment analysis and KEGG pathway enrichment analysis on the differential gene sets. The significance cutoff for GO and KEGG analyses was FDR < 0.05. The GO and KEGG analyses came from the alfalfa-annotated genes (https://modms.lzu.edu.cn/ (accessed on 11 March 2024)).

### 4.5. Co-Expression Network Analysis and Alternative Splicing Analysis

Co-expression network analysis was conducted using the rstatix package for the R language (https://CRAN.R-project.org/package=rstatix, accessed on 10 April 2023). The software rMATS4.0.2 [66] was used to identify alternative splicing (AS) events and analyze differential AS events between the RNA-seq data of SP and NP pistils (with FDR < 0.05).

### 4.6. Real-Time qPCR Analysis

The cDNAs from the SP and NP pistils were synthesized by reverse transcription with HiScript III All-in-one RT SuperMix Perfect for qPCR (Vazyme, Nanjing, China). In total, 15 DEGs were randomly selected from the RNA-seq datasets, and their expression levels were detected using RT-qPCR with Taq Pro Universal SYBR qPCR Master Mix (Vazyme) on a LightCycler 480 Instrument II (Roche, Basel, Switzerland). The program was as follows: a hold at 95 °C for 10 min; 45 cycles of 95 °C for 10 s and 60 °C for 30 s; a melting-curve stage of 95 °C for 15 s and 60 °C for 1 min; and a final heating to 95 °C for 1 s. ACC1 (alfalfa acetyl CoA carboxylase gene) was used as the reference gene [67]. Three biological replicates were performed, and the relative expression level of each gene was calculated via the 2^−ΔΔCt^ method [68]. All primers used are listed in Table S2.

## 5. Conclusions

Self-incompatibility in alfalfa is an elaborate process whose molecular mechanism is poorly understood. In this study, we observed that the pollen tubes germinated on the stigma, grew through the style, and reached the ovarian cavity but were unable to effectively accomplish fertilization. We conducted a transcriptomic analysis of self-pollinated pistils compared with non-pollinated pistils and identified some DEGs that might be involved in SI. GO analysis showed that the DEGs were highly enriched in functions associated with pollen tubes. F-box proteins, serine/threonine protein kinases, CDPKs, bHLHs, bZIPs, and MYB-related family proteins were likely involved in the SI response. Our co-expression network analysis revealed a strong correlation within these DEGs, providing a catalog of candidate genes to enhance our understanding of the molecular regulatory mechanisms of SI in alfalfa.

## Figures and Tables

**Table 1:** Sequencing data statistics of the alfalfa pistil transcriptome.

Sample	Raw Reads (bp)	Clean Reads (bp)	GC (%)	Q20 (%)	Q30 (%)	Total Map (%)
NP_1	42,774,868	42,752,658	41.20	97.80	93.77	93.99
NP_2	39,413,628	39,394,954	40.85	97.91	93.99	93.93
NP_3	39,007,416	38,991,678	40.90	98.23	94.53	94.37
SP_1	47,248,994	46,248,838	41.50	97.81	93.78	93.96
SP_2	49,457,832	47,912,396	40.73	97.83	93.79	93.71
SP_3	40,457,936	39,450,258	41.00	97.81	93.8	93.75

Note: sample: the sample name; raw reads (bp): the total output data, which contains low-quality and spliced reads; clean reads (bp): the effective data after filtering the unqualified reads; GC (%): the GC content; Q20 (%): number of errors per 100 bases; Q30 (%): number of errors per 1000 bases; total map (%): the percentage of reads mapped to the reference genome.

**Table 2:** TFs involved in SI in alfalfa.

Gene ID	Gene Family	Domain	Gene Sample
MS.gene008665	Nin-like	RWP-RK domain-containing protein	*RKD3*
MS.gene010081	GATA	GATA-type zinc finger protein with TIFY domain	*GATA25*
MS.gene013182	bHLH	bHLH family protein	*CKG*
MS.gene014987	Nin-like	RWP-RK domain-containing protein	*RKD3*
MS.gene03941	Nin-like	RWP-RK domain-containing protein	*RKD3*
MS.gene042288	bHLH	LJRHL1-like 1	*LRL1*
MS.gene045066	NAC	NAC family protein	*ATAF1*
MS.gene055646	E2F/DP	E2F transcription factor 3	*E2F3*
MS.gene056509	bZIP	Basic region/leucine zipper transcription factor 16	*BZIP16*
MS.gene05746	Nin-like	RWP-RK domain-containing protein	*RKD3*
MS.gene062584	bZIP	bZIP family protein	*BZIP61*
MS.gene42616	Nin-like	RWP-RK domain-containing protein	*RKD3*
MS.gene69800	bHLH	bHLH family protein	*BHLH10*
MS.gene31282	ARF	Auxin response factor 10	*ARF10*
MS.gene056615	B3	B3 family protein	*B3*
MS.gene005560	ERF	Ethylene-responsive element binding protein	*ERF72*
MS.gene057432	ERF	ERF family protein	*ERF17*
MS.gene54645	HD-ZIP	Homeobox 1	*HB-1*
MS.gene60203	MYB-related	MYB_related family protein	*ATTBP3*

## Data Availability

The original contributions presented in the study are included in the article/Supplementary Material, further inquiries can be directed to the corresponding authors.

## Supplementary Materials

The following supporting information can be downloaded at: https://www.mdpi.com/article/10.3390/plants13060875/s1. Figure S1: Dissected pistils at 48 h after self-pollination; Figure S2: Relative expression of 15 randomly selected genes in RNA-seq in non-pollinated pistils and self-pollinated pistils 24 h after self-pollination; Figure S3: KEGG enrichment analysis of the DEGs in five categories; Figure S4: Expression profiles of crucial candidate TF genes related to SI in alfalfa; Figure S5: Aniline blue staining of pollen tubes (with ovary) 24 h after self-pollination; Table S1: The statistical data of developing ovules at 48 h after self-pollination and cross-pollination; Table S2: The randomly selected sequences subjected to RT-qPCR analysis and their corresponding primers; Table S3: The enriched KEGG pathways that might be related to SI in alfalfa; Table S4: DEGs involved in the SI of alfalfa.
