# Supplementary material for: Transcriptomic Analysis of Self-Incompatibility in Alfalfa
Source: Plants (Basel). 2024 Mar 19;13(6):875. doi: 10.3390/plants13060875 (PMC10975240; doi:10.3390/plants13060875)
Supplement: Supplementary file 1 [file plants-13-00875-s001.zip › plants-2855094-supplementary.pdf]

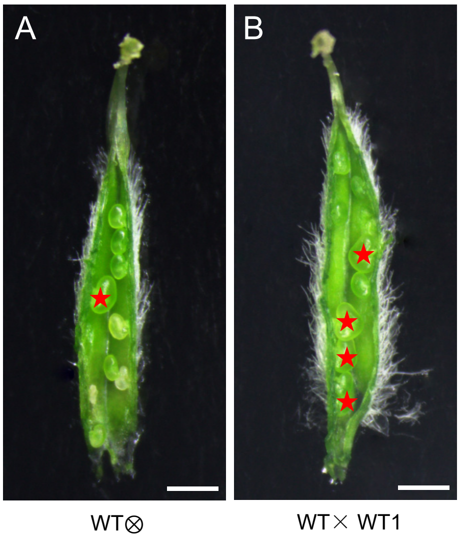

Figure S1. Dissected pistils at 48 h after self-pollination (A) and cross-pollination (B) Bars = 1 mm, The asterisks indicate the developing ovules.

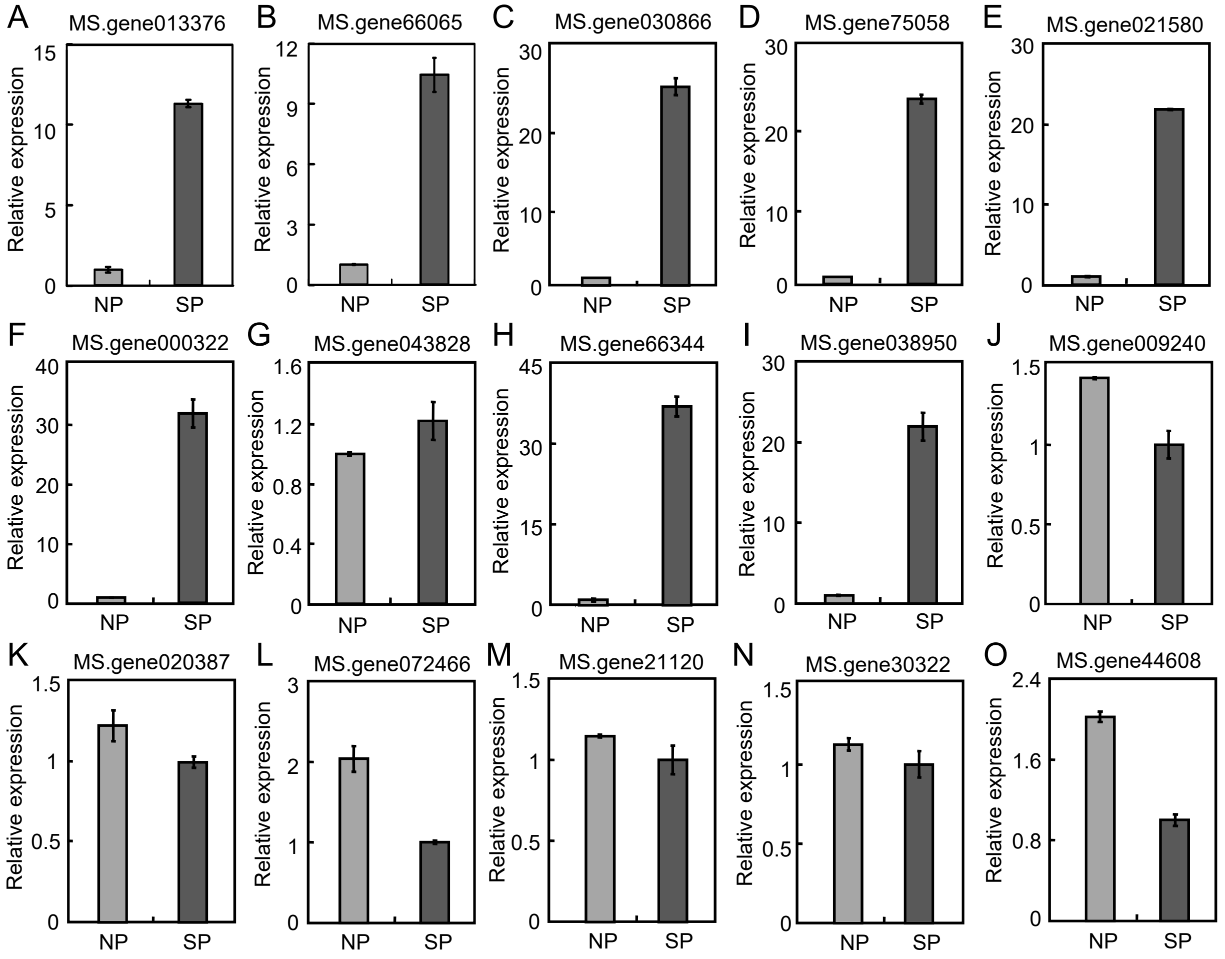

Figure S2. (A) to (O) Relative expression of 15 randomly selected genes in RNA-seq in non-pollinated pistils and self-pollinated pistils 24h after self-pollination. The expression patterns of these genes were consistent with the transcriptomic analysis.

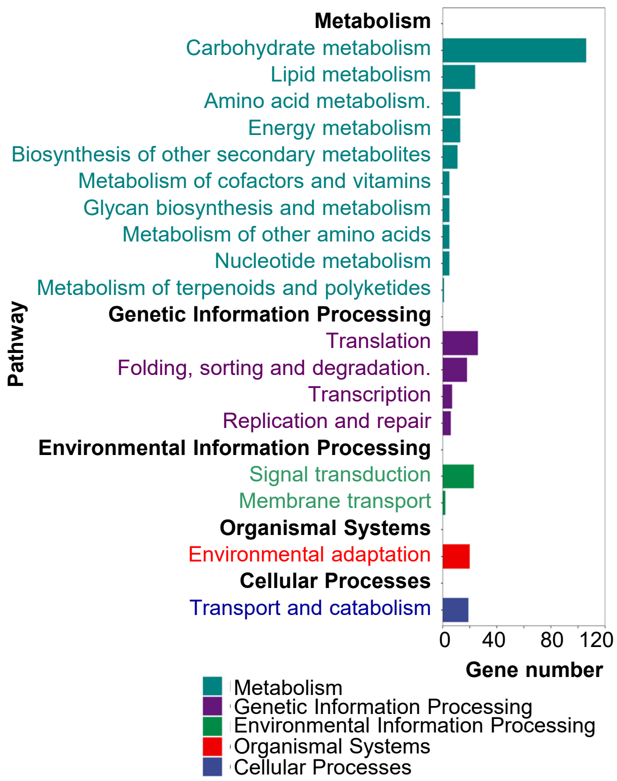

Figure S3. KEGG enrichment analysis of the DEGs in five categories.

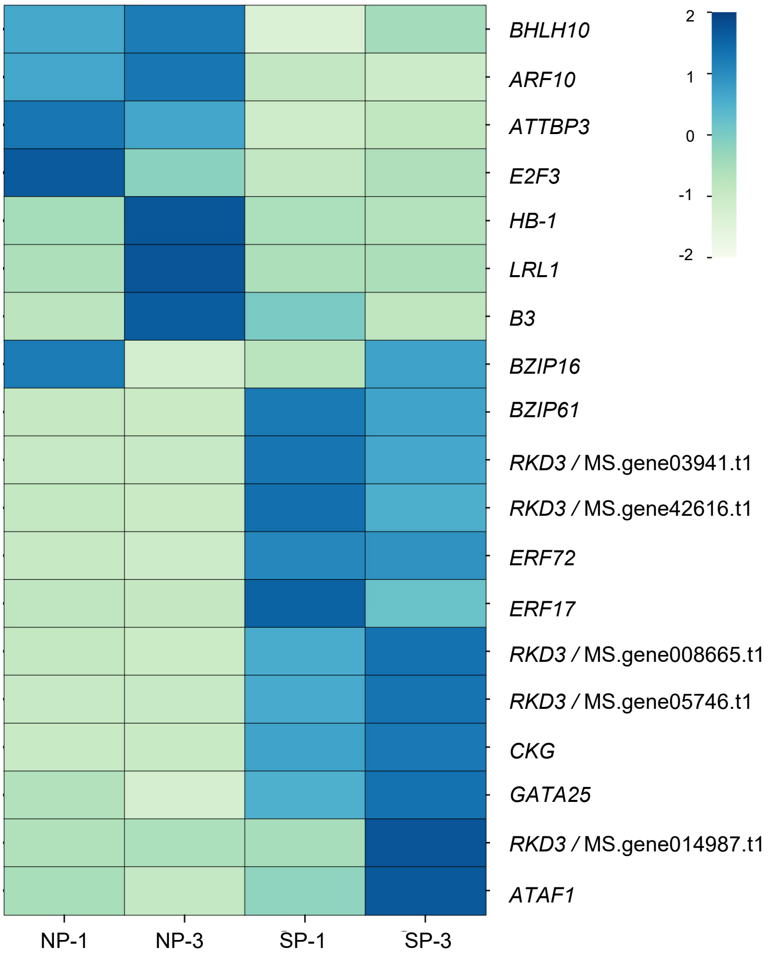

Figure S4. Expression profiles of crucial candidate TF genes related to SI in alfalfa. Pale yellow and blue represent relative decreases and increases in expression, respectively ($\log_2^{FPKM+1}$).

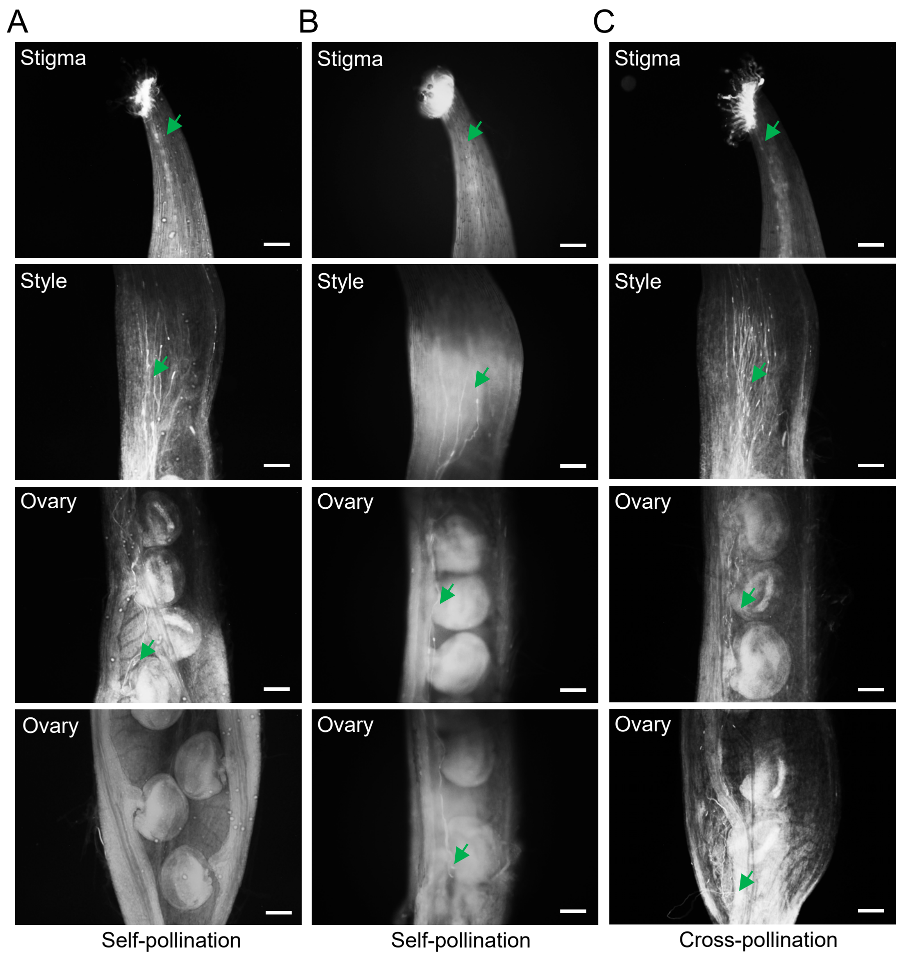

Figure S5. Aniline blue staining of pollen tubes (with ovary) 24 h after self-pollination (A, B) and cross-pollination (C), respectively. Green arrowheads indicate pollen tubes. Bars = 100 μm.

Table S1. The statistical data of developing ovules at 48 h after self-pollination and cross-pollination.

	WT⊗		WT× WT1	
	Total ovules	Developing ovules	Total ovules	Developing ovules
	11	3	10	3
	8	1	10	5
	10	2	9	6
	11	0	9	3
	9	1	10	1
	8	2	10	7
	10	3	10	3
	10	1	8	2
	10	0	11	5
	10	2	11	4
	10	3	11	3
	9	1	10	5
mean	9. 7	1.6	9.9	3.9
SD	0.98	1.08	0.90	1.73

Table S2. The randomly selected sequences subjected to RT-qPCR analysis and their corresponding primers.

Gene name	log_2FoldChange	Primer name	Sequence (5'-3')
MS.gene000322	9.43124951	322-qPCR-F	GCGTGCTCTGAATGTGACAA
		322-qPCR-R	GGTGCCATCTTCAACAACGT
MS.gene013376	8.53728926	13376-qPCR-F	TTGGGATTGGCTGGTAATGC
		13376-qPCR-R	TCCAATCAACATGCTTGCCA
MS.gene021580	10.14372115	21580-qPCR-F	GGCATACTCTTGACCACCCT
		21580-qPCR-R	GTCCATCTCACCCTCACCAA
MS.gene030866	8.632265229	30866-qPCR-F	ACAACGTTAGGAGCAGGGAA
		30866-qPCR-R	GGGCGATTAACCTTGCTGAG
MS.gene038950	10.02934027	38950-qPCR-F	CAGGAACGAGCAAACACCAA
		38950-qPCR-R	CAGTCACGGTAGAATTGGCG
MS.gene043828	9.659001868	43828-qPCR-F	ATCCTCAAGTGCAGTCCCTC
		43828-qPCR-R	TTTGGGTGTTGATGTCTCCG
MS.gene66065	8.595550347	66065-qPCR-F	GACATGCCATCACCAACACA
		66065-qPCR-R	CATGGCTTCATCAACGGCTT
MS.gene66344	10.02737993	66344-qPCR-F	GCCACATTTGCCCTCTTCTC
		66344-qPCR-R	TGATTCCCAACGTGTCACAC
MS.gene75058	9.247972758	75058-qPCR-F	GGCGAGAAACCATGTGATGA
		75058-qPCR-R	ATTCGCTCAACAAGATGCCC
MS.gene009240	-8.444276124	9240-qPCR-F	ATGCAGCCAACATCACCTTG
		9240-qPCR-R	AATAGGTGGATGAGGGCTCC
MS.gene020387	-10.83562464	20387-qPCR-F	AATCTTCAGGGTAGTCGGGC
		20387-qPCR-R	GCTTTGCTGGTTCCATTTGC
MS.gene21120	-8.183796722	21120-qPCR-F	ATCTGGAGGACGCACAATGA
		21120-qPCR-R	CCTGCCACACATCATAAGCC
MS.gene30322	-7.836183848	30322-qPCR-F	CCATTGCAGCATACATAGGACA
		30322-qPCR-R	GCGTGAAATTCCTACTCCACC
MS.gene44608	-8.097665361	44608-qPCR-F	AAAGGAACGCATCTGGAGGA
		44608-qPCR-R	TCCAGCTTTCATAGGCCCAA
MS.gene072466	-8.940955964	72466-qPCR-F	CCTATGGGTTGGCGGAAATG
		72466-qPCR-R	TCAGTGAAGTTCCCCATCCC

Table S3. The enriched KEGG pathways that might be related to SI in alfalfa.

Pathway	Pathway class	Pathway subclass	Description	*p* value
ko00010	Metabolism	Carbohydrate metabolism	Glycolysis / Gluconeogenesis	0.961244734
ko00020	Metabolism	Carbohydrate metabolism	Citrate cycle (TCA cycle)	0.838421643
ko00030	Metabolism	Carbohydrate metabolism	Pentose phosphate pathway	0.7017311
ko00040	Metabolism	Carbohydrate metabolism	Pentose and glucuronate interconversions	5.28E-39
ko00051	Metabolism	Carbohydrate metabolism	Fructose and mannose metabolism	0.807176703
ko00052	Metabolism	Carbohydrate metabolism	Galactose metabolism	0.918618236
ko00053	Metabolism	Carbohydrate metabolism	Ascorbate and aldarate metabolism	1.39E-06
ko00061	Metabolism	Lipid metabolism	Fatty acid biosynthesis	0.830957858
ko00071	Metabolism	Lipid metabolism	Fatty acid degradation	0.843214174
ko00073	Metabolism	Lipid metabolism	Cutin, suberine and wax biosynthesis	0.809332449
ko00100	Metabolism	Lipid metabolism	Steroid biosynthesis	0.013048419
ko00190	Metabolism	Energy metabolism	Oxidative phosphorylation	0.764291477
ko00220	Metabolism	Amino acid metabolism	Arginine biosynthesis	0.802025168
ko00230	Metabolism	Nucleotide metabolism	Purine metabolism	0.871958469
ko00240	Metabolism	Nucleotide metabolism	Pyrimidine metabolism	0.886410909
ko00260	Metabolism	Amino acid metabolism	Glycine, serine and threonine metabolism	0.891871645
ko00261	Metabolism	Biosynthesis of other secondary metabolites	Monobactam biosynthesis	0.129776886
ko00270	Metabolism	Amino acid metabolism	Cysteine and methionine metabolism	0.385624426
ko00280	Metabolism	Amino acid metabolism	Valine, leucine and isoleucine degradation	0.153074214
ko00290	Metabolism	Amino acid metabolism	Valine, leucine and isoleucine biosynthesis	0.009671692
ko00300	Metabolism	Amino acid metabolism	Lysine biosynthesis	0.567669452
ko00400	Metabolism	Amino acid metabolism	Phenylalanine, tyrosine and tryptophan biosynthesis	0.565742075
ko00430	Metabolism	Metabolism of other amino acids	Taurine and hypotaurine metabolism	0.486313901
ko00440	Metabolism	Metabolism of other amino acids	Phosphonate and phosphinate metabolism	0.218704244
ko00450	Metabolism	Metabolism of other amino acids	Selenocompound metabolism	0.526967681
ko00460	Metabolism	Metabolism of other amino acids	Cyanoamino acid metabolism	0.974799551
ko00480	Metabolism	Metabolism of other amino acids	Glutathione metabolism	0.986978685
ko00500	Metabolism	Carbohydrate metabolism	Starch and sucrose metabolism	0.370137402
ko00510	Metabolism	Glycan biosynthesis and metabolism	N-Glycan biosynthesis	0.54840215
ko00513	Metabolism	Glycan biosynthesis and metabolism	Various types of N-glycan biosynthesis	0.416831918
ko00520	Metabolism	Carbohydrate metabolism	Amino sugar and nucleotide sugar metabolism	0.437780772
ko00531	Metabolism	Glycan biosynthesis and metabolism	Glycosaminoglycan degradation	0.034254364
ko00561	Metabolism	Lipid metabolism	Glycerolipid metabolism	0.203677527
ko00562	Metabolism	Carbohydrate metabolism	Inositol phosphate metabolism	0.123166704
ko00564	Metabolism	Lipid metabolism	Glycerophospholipid metabolism	0.006189054
ko00565	Metabolism	Lipid metabolism	Ether lipid metabolism	0.012501572
ko00590	Metabolism	Lipid metabolism	Arachidonic acid metabolism	0.259524391
ko00591	Metabolism	Lipid metabolism	Linoleic acid metabolism	0.393086613
ko00592	Metabolism	Lipid metabolism	alpha-Linolenic acid metabolism	0.017973497
ko00600	Metabolism	Lipid metabolism	Sphingolipid metabolism	0.444754945
ko00620	Metabolism	Carbohydrate metabolism	Pyruvate metabolism	0.697077399
ko00630	Metabolism	Carbohydrate metabolism	Glyoxylate and dicarboxylate metabolism	0.848571144
ko00660	Metabolism	Carbohydrate metabolism	C5-Branched dibasic acid metabolism	0.312044722
ko00710	Metabolism	Energy metabolism	Carbon fixation in photosynthetic organisms	0.951308967
ko00770	Metabolism	Metabolism of cofactors and vitamins	Pantothenate and CoA biosynthesis	0.105313994
ko00860	Metabolism	Metabolism of cofactors and vitamins	Porphyrin metabolism	0.930022123
ko00900	Metabolism	Metabolism of terpenoids and polyketides	Terpenoid backbone biosynthesis	0.847865076
ko00901	Metabolism	Biosynthesis of other secondary metabolites	Indole alkaloid biosynthesis	0.252466934
ko00910	Metabolism	Energy metabolism	Nitrogen metabolism	0.18394727
ko00920	Metabolism	Energy metabolism	Sulfur metabolism	0.182088663
ko00940	Metabolism	Biosynthesis of other secondary metabolites	Phenylpropanoid biosynthesis	0.982424699
ko00943	Metabolism	Biosynthesis of other secondary metabolites	Isoflavonoid biosynthesis	0.846715398
ko00966	Metabolism	Biosynthesis of other secondary metabolites	Glucosinolate biosynthesis	0.730564507

Continued

Table S3. The enriched KEGG pathways that might be related to SI in alfalfa

Pathway	Pathway class	Pathway subclass	Description	*p* value
ko00970	Genetic Information Processing	Translation	Aminoacyl-tRNA biosynthesis	0.971334653
ko02010	Environmental Information Processing	Membrane transport	ABC transporters	0.882445449
ko03008	Genetic Information Processing	Translation	Ribosome biogenesis in eukaryotes	0.660531836
ko03010	Genetic Information Processing	Translation	Ribosome	0.148477398
ko03013	Genetic Information Processing	Translation	Nucleocytoplasmic transport	0.990360695
ko03015	Genetic Information Processing	Translation	mRNA surveillance pathway	0.745609207
ko03018	Genetic Information Processing	Folding, sorting and degradation	RNA degradation	0.808587675
ko03020	Genetic Information Processing	Transcription	RNA polymerase	0.919230077
ko03030	Genetic Information Processing	Replication and repair	DNA replication	0.999938895
ko03040	Genetic Information Processing	Transcription	Spliceosome	0.981486808
ko03050	Genetic Information Processing	Folding, sorting and degradation	Proteasome	0.539467558
ko03410	Genetic Information Processing	Replication and repair	Base excision repair	0.910231247
ko03420	Genetic Information Processing	Replication and repair	Nucleotide excision repair	0.999997773
ko03430	Genetic Information Processing	Replication and repair	Mismatch repair	0.999993383
ko03440	Genetic Information Processing	Replication and repair	Homologous recombination	0.999288456
ko04016	Environmental Information Processing	Signal transduction	MAPK signaling pathway - plant	0.947414312
ko04070	Environmental Information Processing	Signal transduction	Phosphatidylinositol signaling system	0.161747581
ko04075	Environmental Information Processing	Signal transduction	Plant hormone signal transduction	0.45490642
ko04120	Genetic Information Processing	Folding, sorting and degradation	Ubiquitin mediated proteolysis	0.999647294
ko04130	Genetic Information Processing	Folding, sorting and degradation	SNARE interactions in vesicular transport	0.051601555
ko04141	Genetic Information Processing	Folding, sorting and degradation	Protein processing in endoplasmic reticulum	0.998207156
ko04144	Cellular Processes	Transport and catabolism	Endocytosis	0.284667919
ko04145	Cellular Processes	Transport and catabolism	Phagosome	0.513984045
ko04146	Cellular Processes	Transport and catabolism	Peroxisome	0.887920802
ko04626	Organismal Systems	Environmental adaptation	Plant-pathogen interaction	0.113339518

Table S4. DEGs involved in the SI of alfalfa.

Gene ID	Annotation
MS.gene03476.t1	F-box protein
MS.gene054335.t1	F-box protein
MS.gene42062.t1	F-box protein
MS.gene57177.t1	F-box-like WD repeat-containing protein
MS.gene74882.t1	Serine threonine-protein kinase
MS.gene07587.t1	Serine threonine-protein kinase
MS.gene005037.t1	Serine threonine-protein kinase
MS.gene070934.t1	Serine threonine-protein kinase
MS.gene54823.t1	Serine threonine-protein kinase
MS.gene065848.t1	Serine threonine-protein kinase
MS.gene067504.t1	LRR receptor-like serine threonine-protein kinase
MS.gene00953.t1	Receptor-like cytosolic serine threonine-protein kinase
MS.gene03081.t1	LRR receptor-like serine threonine-protein kinase
MS.gene051225.t1	Serine threonine-protein kinase
MS.gene048989.t1	LRR receptor-like serine threonine-protein kinase
MS.gene070324.t1	LRR receptor-like serine threonine-protein kinase
MS.gene53398.t1	LRR receptor-like serine threonine-protein kinase
MS.gene39640.t1	Receptor-like serine threonine-protein kinase
MS.gene068586.t1	Receptor-like cytosolic serine threonine-protein kinase RBK1
MS.gene001042.t1	Serine threonine-protein kinase
MS.gene68843.t1	LRR receptor-like serine threonine-protein kinase
MS.gene031103.t1	Serine threonine-protein kinase
MS.gene75108.t1	LRR receptor-like serine threonine-protein kinase
MS.gene86052.t1	Serine threonine-protein kinase
MS.gene66283.t1	LRR receptor-like serine threonine-protein kinase
MS.gene024491.t1	Serine threonine-protein kinase
MS.gene45217.t1	Serine threonine-protein kinase
MS.gene072587.t1	Serine threonine-protein kinase
MS.gene22844.t1	Serine threonine-protein kinase
MS.gene49546.t1	Serine threonine-protein kinase
MS.gene008945.t1	Receptor-like cytosolic serine threonine-protein kinase RBK1
MS.gene84738.t1	Serine threonine-protein kinase
MS.gene89935.t1	Calcium-dependent protein kinase
MS.gene056364.t1	Calcium-dependent protein kinase
MS.gene030537.t1	Calcium-dependent protein kinase
MS.gene28353.t1	Calcium-dependent protein kinase
MS.gene54572.t1	Calcium-dependent protein kinase
MS.gene003898.t1	Calcium-dependent protein kinase
MS.gene016961.t1	Calcium-dependent protein kinase
MS.gene47650.t1	Calcium-dependent protein kinase
MS.gene70857.t1	Calcium-dependent protein kinase
